# Carbon Nanofiber-Based Functional Nanomaterials for Sensor Applications

**DOI:** 10.3390/nano9071045

**Published:** 2019-07-22

**Authors:** Zhuqing Wang, Shasha Wu, Jian Wang, Along Yu, Gang Wei

**Affiliations:** 1 AnHui Provice Key Laboratory of Optoelectronic and Magnetism Functional Materials, Anqing Normal University, Anqing 246011, China; 2 College of Chemistry and Chemical Engineering, Qingdao University, Qingdao 266077, China; 3 Hybrid Materials Interfaces Group, Faculty of Production Engineering and Center for Environmental Research and Sustainable technology (UFT), University of Bremen, D-28359 Bremen, Germany

**Keywords:** carbon nanofibers, nanoparticles, electrospinning, hybrid nanomaterials, sensor

## Abstract

Carbon nanofibers (CNFs) exhibit great potentials in the fields of materials science, biomedicine, tissue engineering, catalysis, energy, environmental science, and analytical science due to their unique physical and chemical properties. Usually, CNFs with flat, mesoporous, and porous surfaces can be synthesized by chemical vapor deposition and electrospinning techniques with subsequent chemical treatment. Meanwhile, the surfaces of CNFs are easy to modify with various materials to extend the applications of CNF-based hybrid nanomaterials in multiple fields. In this review, we focus on the design, synthesis, and sensor applications of CNF-based functional nanomaterials. The fabrication strategies of CNF-based functional nanomaterials by adding metallic nanoparticles (NPs), metal oxide NPs, alloy, silica, polymers, and others into CNFs are introduced and discussed. In addition, the sensor applications of CNF-based nanomaterials for detecting gas, strain, pressure, small molecule, and biomacromolecules are demonstrated in detail. This work will be beneficial for the readers to understand the strategies for fabricating various CNF-based nanomaterials, and explore new applications in energy, catalysis, and environmental science.

## 1. Introduction

With the development of nanotechnology and material science, many kinds of zero-dimensional (0D) to three-dimensional (3D) materials have been created for sensor applications [1,2,3,4,5], in which the one-dimensional (1D) nanomaterials exhibited promising potential [6]. It is well known that 1D architectures could provide shortened pathways for the transfer of electrons and facilitate the penetration of electrolyte along the longitudinal axis of nanofiber/nanowire [7,8], and therefore causing improved sensing performance.

Carbon nanotubes (CNTs), as one of the widely used 1D nanomaterials, have been previously utilized for the fabrication of various high-performance sensors and biosensors due to the unique mechanical, electrical, and magnetic properties of CNTs [9,10]. In addition, the high surface area and high adsorption ability towards various molecules/biomolecule of CNTs make CNTs very good candidates to fabricate chemical and biological sensors with high sensitivity and selectivity.

Besides CNTs, carbon nanofibers (CNFs) have also been widely studied due to their unique chemical and physical properties and similar structure to fullerenes and CNTs [11,12]. CNTs are hollow with a graphite layer parallel to the axis of the inner tube. The graphite layers of CNFs often form an angle with the axis of the inner tube, and the interior thereof may be hollow or solid. The diameters of CNTs are usually less than 100 nm, while the diameter of CNF is in the range of 10 to 500 nm and the length can reach 10 μm. Meanwhile, CNFs have exclusively basal graphite planes and edge planes, exhibiting high potentials for their surface modification or functionalization to create functional hybrid CNF-based nanomaterials, which have been applied in the fields of biomedicine [13], tissue engineering [14], nanodevices [11], sensors [15,16], energy [17], and environmental science [18]. Previously, several reviews on the synthesis and applications of CNFs and CNF-based materials have been released [19,20,21,22,23]. For instance, Zhang et al. demonstrated the potential strategies for creating CNFs with electrospinning and then summarized their applications in biomedicine, sensor, energy, and environmental fields [19]. Feng et al. summarized the synthesis, properties, and applications of CNFs and CNF-based composites, in which the applications of CNF materials in electrical devices, batteries, and supercapacitors were introduced in detail [20]. Zhang and co-workers provided recent advances in the electrospun synthesis and electrochemical energy storage application of CNFs [21].

In this review, we focus on the preparation of CNF-based nanomaterials for sensor applications. In the second part, we introduced the synthesis strategies of CNFs via chemical vapor deposition and electrospinning techniques, and in the third part we demonstrated the design and synthesis of CNF-based nanomaterials by the functionalization of pure CNFs with metallic nanoparticles (NPs), metal oxide NPs, alloy NPs, silica, and polymers. In the fourth part, the sensor applications of CNF-based nanomaterials towards gas, strain, pressure, small molecules, and biomacromolecules are introduced and discussed. Finally, the conclusions and outlooks for the synthesis and applications of CNF-based nanomaterials are given. It is expected that this work will be helpful for the readers to understand the sensing mechanisms of CNF-based sensors and develop new CNF-based nanomaterials for the applications besides sensors.

## 2. Synthesis of Carbon Nanofibers (CNFs)

**Figure 1:**
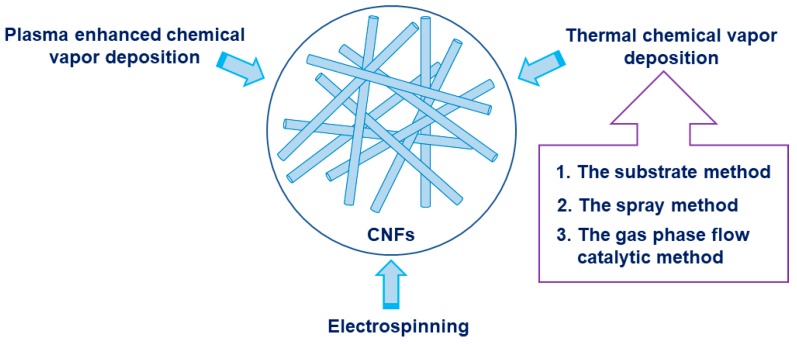
Typical method for synthesizing Carbon nanofibers (CNFs).

CNFs have large specific surface area, small number of defects, large aspect ratio, low density, high specific modulus and strength, high electrical and thermal conductivity, etc., and therefore have broad application prospects in the fields of storage, electrochemistry, adsorbent, and sensing [11,24,25,26,27]. At present, methods for preparing CNFs mainly include thermal chemical vapor deposition, plasma enhanced chemical vapor deposition, and electrospinning [28], as shown in Figure 1.

### 2.1. Thermal Chemical Vapor Deposition

The chemical vapor deposition method is a method for synthesizing CNFs by thermally decomposing a low-cost hydrocarbon compound on a metal catalyst at a constant temperature (500–1000 °C) [20,29]. The thermal chemical vapor deposition method can be classified into the following three types according to the manner of the catalyst added or present: The substrate method, the spray method, and the gas phase flow catalytic method.

#### 2.1.1. The Substrate Method

The substrate method utilizes ceramic or SiO_2_ fibers as substrate to uniformly disperse nanosized catalyst particles (such as Fe, Co, Ni, and other transition metals) on its surface. The hydrocarbon gas is pyrolyzed on the surface of the catalyst, and carbon is deposited and grown to obtain nanoscale carbon fibers. Enrique et al. fabricated high purity CNFs on the ceramic substrate by using CH_4_/H_2_ (or C_2_H_6_/H_2_) as the carbon source and Ni as the catalyst at 873 K, and explored the influence of conditions (different carbon sources, temperatures, etc.,) on the layer thickness, uniformity, and porosity of CNFs [30].

The substrate method can prepare CNFs of relatively high purity, but the CNFs can only grow on the substrate dispersed with catalyst particles. Since nanoscale catalyst preparation is difficult and the product and catalyst cannot be separated in time, it is difficult to achieve large-scale continuous production of CNFs.

#### 2.1.2. The Spray Method

The spraying method is to mix the catalyst in a liquid organic substance such as benzene, and then spray the mixed solution containing the catalyst into a high temperature reaction chamber to prepare CNFs. The continuous injection of the catalyst can be realized by growing the carbon fiber by the spray method, which provides favorable conditions for industrial continuous production [31,32]. However, the uneven distribution of the catalyst particles during the spraying process and the ratio of the hydrocarbon gas to the hydrocarbon gas are difficult to control, resulting in a small proportion of the CNFs produced by this method, and a certain amount of carbon black is formed.

#### 2.1.3. The Gas Phase Flow Catalytic Method

The gas phase flow catalysis method directly heats the catalyst precursor, and introduces it into the reaction chamber together with the hydrocarbon gas in the form of gas. The catalyst and the hydrocarbon gas are decomposed of different temperature zones, and the decomposed catalyst atoms are gradually aggregated into the nanoscale particles and then the CNFs produced on the nanoscale catalyst particles [33,34]. Since the catalyst particles decomposed from the organic compound can be distributed in a three-dimensional space, and the amount of volatilization of the catalyst can be directly controlled, the yield per unit time of this method is large and continuous production is possible.

### 2.2. Plasma-Enhanced Chemical Vapor Deposition (PECVD)

The plasma contains a large amount of high-energy electrons that can provide the activation energy required for the chemical vapor deposition process. The collision of electrons with gas phase molecules can promote the decomposition, compounding, excitation, and ionization processes of gas molecules, and generate various chemical groups with high activity [35,36,37]. While the plasma enhanced chemical vapor deposition method can produce aligned CNFs, the cost of this method is high, the production efficiency is low, and the process is difficult to control.

### 2.3. Electrospinning

Electrospinning technology first appeared in the 1930s. It has renewed interest in recent years and was used to prepare CNFs. It is also the only method to produce continuous CNFs [19,30,38,39,40,41,42]. In the electrospinning process, first the polymer solution or melt is charged with thousands to tens of volts of static electricity. The charged polymer forms a Taylor cone at the spinning port under the action of an electric field. When the electric force exceeds the internal tension of the spinning solution, the Taylor cone is drafted and accelerated. The moving jet is gradually drafted and thinned. Due to its extremely fast rate of motion, the fibers ultimately deposited on the collecting plate are nanoscale, forming a fibrous mat similar to a nonwoven fabric. Then, the fiber mat is pre-oxidized in air and carbonized in a nitrogen atmosphere to finally obtain CNFs. 

Compared with other nanofiber manufacturing methods, the electrospinning method has the following advantages: (1) Electrospinning usually uses voltages of several thousand volts or more, but the current used is small, so that energy consumption is small; and (2) a nanofiber nonwoven fabric can be directly produced. By electrospinning, the nanofibers can be made into a nonwoven fabric in a two-dimensional expanded form, so that no further processing is required after spinning. In particular, the development of multi-nozzle spinning technology has increased the production of nanofiber nonwovens and improved production efficiency; (3) it can be spun at room temperature. The electrospinning method allows spinning at room temperature, so that a solution containing a compound having poor thermal stability can also be spun; (4) a wide range of raw materials. Thus far, there have been reports on the use of synthetic polymers such as polyester and polyamide, and natural high molecular substances such as collagen, silk, and DNA as raw materials to prepare nanofibers by electrospinning.

## 3. Design and Synthesis of CNF-Based Nanomaterials

In recent years, with the rapid development of nanofabrication technology, more and more carbon-based nanomaterials have been used as sensors for detecting different target molecules [43,44,45]. Depending on the type of material being loaded, we can classify the carbon-based nanofibers used as sensors into five types: Pure CNFs, CNFs loaded with metal NPs, CNFs loaded with metal oxides, CNFs loaded with metal alloys, and others.

### 3.1. Pure CNFs

**Figure 2:**
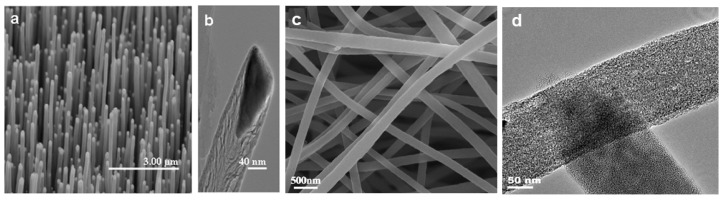
(**a**) SEM image of vertically aligned CNF array, (**b**) TEM image of a stacked cone morphology of CNFs, (**c**) SEM image of pure CNFs, and (**d**) TEM image of a single CNF. Pictures (**a**) and (**b**) were reprinted with permission from Reference [49]. Copyright American Chemical Society, 2013. Pictures (**c**) and (**d**) were reprinted with permission from Ref. [50]. Copyright Elsevier, 2011.

Due to their high specific surface area and good electrocatalytic ability towards the oxidation of specific organic matter, pure CNFs are commonly used to detect small molecules, viruses, proteins, and nucleic acids in food quality control and clinical analysis. For example, Yue et al. reported mesoporous CNF-modified pyrolytic graphite electrode for the simultaneous determination of uric acid, ascorbic acid, and dopamine [46]. Koehn et al. prepared a vertically aligned CNF electrode array by the PECVD method, and then integrated the CNF array with the wireless instantaneous neurotransmitter sensor system to detect dopamine by fast scan cyclic voltammetry [47]. Rand and coworkers developed a biosensor based on vertically aligned CNFs for the simultaneous detection of serotonin and dopamine in the presence of excess ascorbic acid [48]. Periyakaruppan et al. reported similar CNFs based nanoelectrode arrays for label-free detecting cardiac troponin-I in the early diagnosis of myocardial infarction (Figure 2a,b) [49].

Tang et al. directly modified electrospun CNFs onto carbon paste electrode (CPE) for the electrochemical detection of xanthine, L-Tryptophan, L-tyrosine, and L-cysteine without any enzyme or medium, respectively (Figure 2c,d) [50,51]. The CNFs-modified CPE showed high electrocatalytic activity and fast amperometric response towards the oxidation of the xanthine and three amino acids. Guo and coworkers reported similar electrospun CNFs-modified CPE for simultaneous determination of catechol and hydroquinone in lake water samples [52].

### 3.2. CNFs Modified with Metal NPs

**Figure 3:**
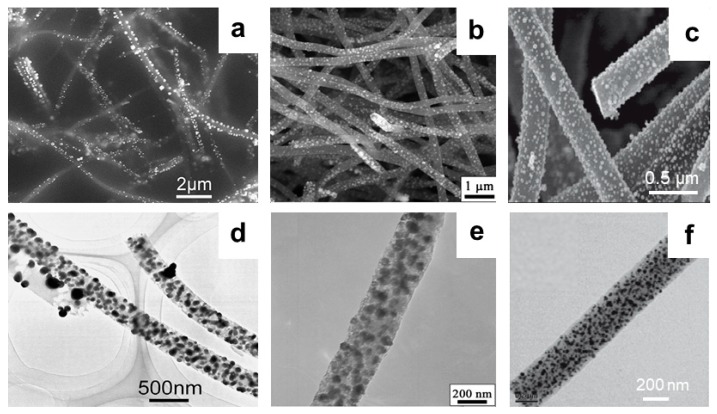
SEM images of (**a**) Pd NPs-decorated CNFs, (**b**) Ni NPs-decorated CNFs and (**c**) Rh NPs-decorated CNFs. TEM images of (**d**) Pd NPs-decorated CNFs, (**e**) Ni NPs-decorated CNFs, and (**f**) Rh NPs-decorated CNFs. Pictures (**a**) and (**d**) were reprinted with permission from Reference [58]. Copyright Elsevier, 2008. Pictures (**b**) and (**e**) were reprinted with permission from Reference [63]. Copyright Elsevier, 2009. Pictures (**c**) and (**f**) were reprinted with permission from Reference [61]. Copyright Elsevier, 2010.

Since the conductivity of the metal NPs and their high electrochemical activity toward the target substance can effectively reduce the overpotential, and they can be embedded in the defect sites of the CNFs to improve the sensitivity and anti-interference ability of the sensor [53,54,55,56]. Huang et al. prepared a Pd NPs-decorated CNFs sensor for detecting H_2_O_2_ and nicotinamide adenine dinucleotide (NADH) [57] (Figure 3a,d). This Pd NPs-loaded CNFs modified electrode can also be used for simultaneously detecting dopamine, uric acid, and ascorbic acid [58]. On the other hand, Liu and coworkers modified Pd NP-loaded CNFs onto the carbon paste electrode for efficient detection of oxalic acid [59]. Claramunt et al. prepared an efficient gas sensor by modifing Au NPs onto CNFs [60]. Hu et al. developed a Rh NP-decorated CNFs sensor for the detection of hydrazine [61] (Figure 3c,f). Fu et al. modified Cu NP-loaded CNFs composite onto the glassy carbon electrode for the detection of catechol [62]. Liu et al. and Rathod et al. modified Ni and Pt NPs onto CNFs, respectively (Figure 3b,e). Additionally, the as-prepared composites can be used for non-enzymatic glucose sensing [63,64]. In addition, the loaded metal NPs can form a more sparse conductive network inside the nanocomposite, which can enhance the electrical conductivity of the CNFs, making the composite highly sensitive to stress. Hu et al. synthesized a composite material for a piezoresistive strain sensor consisting of Ag NPs-coated CNFs with an epoxy resin, which shows an extremely high sensitivity to stress changes [65].

### 3.3. CNFs Modified with Metal Oxides

Since some acid gases and organic gases can cause changes in the electrical resistance of metal oxide-decorated CNFs, metal oxide-decorated CNFs can be used for the detection of specific acid gas and organic gas. Lee and coworkers fabricated ZnO/SnO_2_ nanonodules-decorated CNFs for dimethyl methylphosphonate gas detection by single nozzle co-electrospinning using two phase-separated polymer solutions [66]. Later, this group modified WO_3_ nanonodule to the surface of CNFs for the detection of NO_2_ gas using the same method, and found that the sensitivity of the WO_3_ nanonodule-decorated CNFs increased the amount of the decorated WO_3_ on the CNFs surface [67].

**Figure 4:**
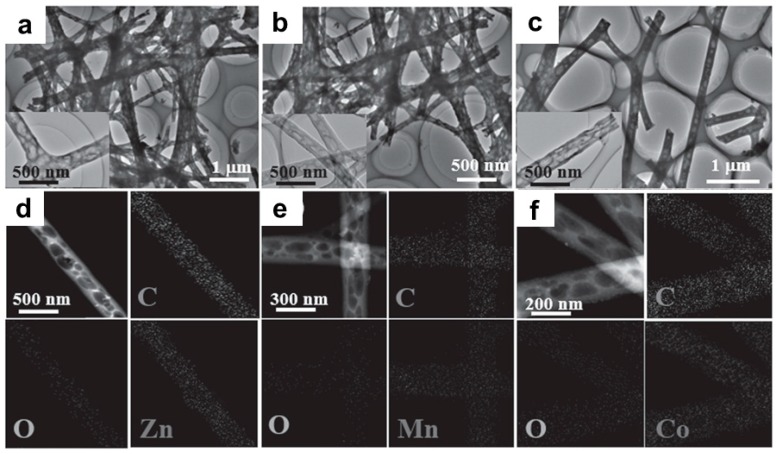
TEM and the corresponding elemental mapping images of (**a**,**d**) ZnO/CNFs, (**b**,**e**) MnO/CNFs, and (**c**,**f**) CoO/CNFs. Reprinted with permission from Reference [69]. Copyright Wiley-VCH, 2016.

Hu and co-workers demonstrated the electrospun preparation of mesoporous MnO_2_ and Mn_3_O_4_ NPs-decorated CNFs, and found that the fabricated hybrid CNFs have a diameter of 200–300 nm with high surface area [68]. In another case, Xia and co-workers reported the general synthesis of ultrafine transition metal oxide (Zn, Mn, and Co) NPs-embedded porous CNFs via a facile electrospinning strategy, following through the calcination process [69]. As shown in Figure 4, there are abundant interconnected pores distributed in the ZnO/CNFs, MnO/CNFs, and CoO/CNFs, and the Zn, Mn, and Co elements are homogeneously distributed inside the porous CNFs, respectively.

### 3.4. CNFs Modified with Alloys

**Figure 5:**
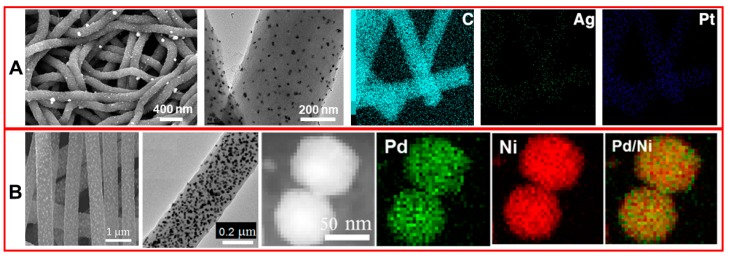
SEM, TEM and the elemental mapping images of (**A**) Ag-Pt alloy NPs-decorated CNFs and (**B**) Pd-Ni alloy NPs-decorated CNFs. Pictures (**A**) were reprinted with permission from Reference [70]. Copyright American Chemical Society, 2014. Pictures (**B**) were reprinted with permission from Reference [71]. Copyright American Chemical Society, 2014.

Compared with single-metal NPs, metals alloy exhibit superior electrocatalysis due to their binary structure interface synergy, which makes metals alloy NPs-modified CNF sensors exhibit stronger anode peak potential and redox current [32]. Huang et al. prepared Ag-Pt alloy NPs by the NaBH_4_ reduction method and modified them onto electrospun CNFs for the selective detection of dopamine (Figure 5A) [70]. Guo and coworkers synthesized Pd-Ni alloy NP/CNFs composite by the simple method involving electrospinning of precursor polyacrylonitrile/Pd(acac)_2_/Ni(acac)_2_ and subsequent thermal process to reduce metals and carbonize polyacrylonitrile (Figure 5B). The as-prepared Pd-Ni alloy NP/CNFs composite significantly improved electrocatalytic activity for sugar oxidation, and Pd-Ni alloy NP/CNFs based electrode can be used for sugar detection in flow systems [71]. Li et al. fabricated a series of MCo (M = Fe, Cu, Mn, and Ni) alloy NPs-decorated CNFs by electrospinning and thermal treatment process, and found that the CuCo alloy NPs doped-CNFs exhibit the best detection efficiency for glucose in human serum samples [72]. 

### 3.5. CNFs Modified with Silica and Polymers

**Figure 6:**
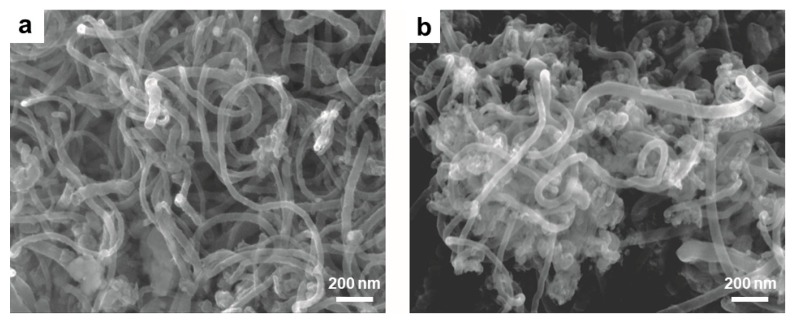
SEM images of (**a**) CNFs and (**b**) silica/CNFs composite. Reprinted with permission from Reference [74]. Copyright American Chemical Society, 2008.

In addition, some other materials such as silica, polyurethanes, polydimethylsiloxane, nafion, etc., are also used to modify CNFs for sensing [73]. For example, Vamvakaki et al. used biomimetically synthesized silica modified CNFs for the detection of acetylcholinesterase, and the fabricated silica/CNF composite shows an operational lifetime of more than 3.5 months under continuous polarization (Figure 6) [74]. Lu and coworkers modified hemoglobin to CNFs with the help of Nafion membrane, and the prepared CNFs-based composite can mediator-free detect H_2_O_2_ [75]. Zhu et al. prepared an elastomer/CNF strain sensing composite for detecting tensile forces [76]. Baeza and coworkers embed CNFs in cement for strain and damage detection [77]. Azhari et al. embed CNFs and carbon nanotubes in cement for piezoresisitive sensing [78]. Tallman et al. embed CNFs in polyurethane for tactile imaging and distributed strain sensing, and found that the piezoresistive response of CNFs/polyurethane nanocomposites depends strongly on the nanofiller volume fraction [79]. The sensitivity of the CNFs/polyurethane nanocomposites increased with decreasing CNFs volume fraction.

## 4. Sensor Applications of CNF-Based Nanomaterials

The higher surface area of CNFs can adsorb relatively more target molecules. In addition, CNFs also have good electron transfer ability. These characteristics make CNFs-based nanomaterials have broad prospects in chemical sensing [80,81,82,83]. According to the type and nature of the target substances, we mainly introduce the application of CNFs-based nanomaterials as sensors in the following four aspects.

### 4.1. Gas Sensors

Li and coworkers prepared one-dimensional CNFs composed of graphitic nanorolls using a simple electrospinning-assisted solid-phase graphitization method, the as-prepared graphitic CNFs exhibit sensitivity to H_2_, CO, CH_4_, and ethanol gases at room temperature, and the detection limit for CO gas is as low as 50 ppm [84]. Zhang et al. reported a H_2_S sensor based on ZnO-CNFs composites, the as-prepared H_2_S sensor showed a linear response in the range of 50–102 ppm of H_2_S [85]. Claramunt et al. deposited metal alloy NPs-decorated CNFs on Kapton for the detection of NH_3_ [60]. The results show that the sensitivity of Au and Pd NPs-decorated CNFs to NH_3_ can be improved by controlling the percentage of Au and Pd. Moreover, the response time of the sensor is up to 5 minutes within 110–120 °C. However, when compared with the spectroscopic sensor such as mid-infrared sensor and quartz-enhanced photoacoustic sensor [86,87,88,89,90], which have the advantages of rapid detection at room temperature without any reagent, the operation temperature of Au, and Pd NPs-decorated CNFs is much higher.

**Figure 7:**
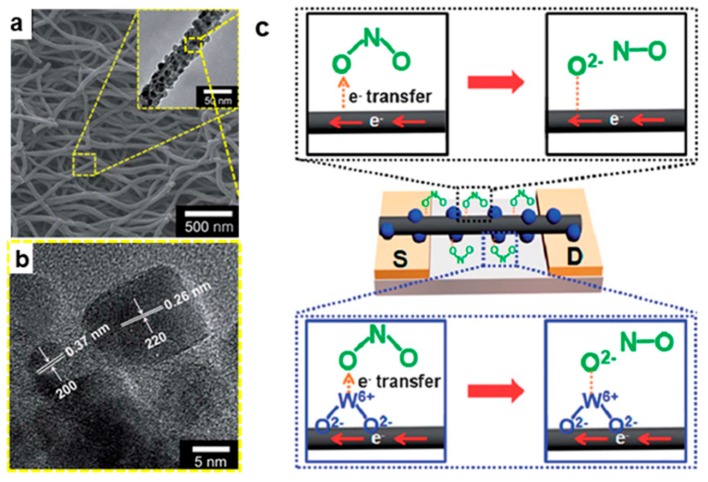
(**a**) SEM and TEM (inset) images of the WO_3_ nanomodule-decorated CNFs; (**b**) High resolution transmission electron microscope (HRTEM) image of the WO_3_ nanomodule-decorated CNFs; and (**c**) NO_2_ gas detection mechanism of the WO_3_ nanomodule-decorated CNFs. Reprinted with permission from Reference [67]. Copyright the Royal Society Chemistry, 2013.

In order to reduce the detection temperature, Lee et al. modified WO_3_ nanonodules onto the CNFs, and the prepared WO_3_ nanomodule-decorated CNFs not only provides a higher sensing surface area, but also WO^2+^ on the surface of the material can combine with the O^2−^ of NO_2_, realizing the detection of NO_2_ gas at room temperature, and the detection limit for NO_2_ reach 1 ppm (Figure 7) [67].

### 4.2. Strain/Pressure Sensors.

**Figure 8:**
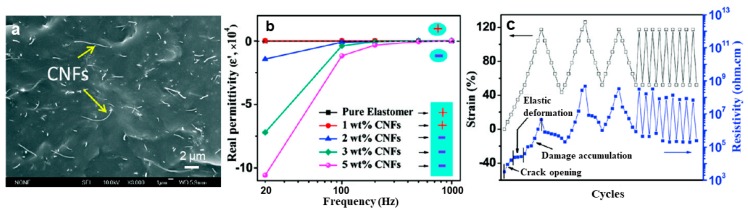
(**a**) SEM image of the 5 wt% CNFs/Vistamaxx 6202FL polymer nanocomposite; (**b**) real permittivity of the CNFs/Vistamaxx 6202FL polymer nanocomposite in the frequency range of 20–1000; and (**c**) cyclic strain applied to specimen and the instantaneous response of resistivity with strain of the 5 wt% CNFs/Vistamaxx 6202FL polymer nanocomposite. Reprinted with permission from Reference [76]. Copyright American Chemical Society, 2011.

Conventional micro-electro mechanical system (MEMS) pressure sensors such as silicon piezoresistive pressure sensor and silicon capacitive pressure sensor have the advantages of high measurement accuracy, low power consumption, and low cost, but perform poorly in high-intensity piezoresistive measurements. Due to its low cost, electrical conductivity, and potentially enhanced mechanical properties such as fracture toughness and strain capacity, CNFs are also commonly used for material structure health monitoring [91,92,93,94,95]. Zhu and coworkers used vistamaxx 6202FL (ethylene content 15 wt%, propylene 85%) as the hosting polymer matrix to fabricate conductive polymer nanocomposites reinforced with CNFs via the solvent-assisted casting method. The as-prepared electrically conductive polymer nanocomposite can be utilized as strain sensors with large mechanical deformation (Figure 8a,b). The resistivity is reversibly changed by 10^2^–10^3^ times upon stretching to 120% strain and recovery to 40% strain (Figure 8c) [76]. 

Azhari et al. prepared a conductive cement-based piezoresistive sensor by mixing 15% CNFs and 1% carbon nanotubes. The sensor is more accurate and repeatable than traditional cement-based sensors, with load amplitudes up to 30 kN and the gauge factor is about 445 [78]. Bazea et al. synthesized a CNF and cement composite to measure strains on the surface of a structural element, and found that the CNF cement-based composite with a gauge factor of 190 can be obtained by adding 2 wt% CNFs to cement [77]. Hu and coworkers fabricated a resistance-type strain sensor by using Ag-coated CNFs and epoxy. The as-prepared Ag-coated CNFs/epoxy composite shows higher strain sensitivity and better conductivity than that of CNFs without Ag-coating, and has a gauge factor of 155, this value is ~80 times higher than that in a metal-foil strain gauge [65]. In the application of CNFs/polyurethane nanocomposite for tactile imaging and distributed strain sensing, Tallman et al. found that the piezoresistive response is most sensitive to strain changes when the CNFs filling volume fraction is 12.5%–15%. When the CNFs filling volume fraction is 7.5%, there is a region in which the conductivity changes the most in the tactile imaging [79]. Yan and coworkers fabricated a flexible strain sensor by using carbon/graphene composites nanofiber yarn/thermoplastic polyurethane, this strain sensor shows a high level of stability during 300 stretching relaxation, and the average gauge factor value is more than 1700 under an applied strain of 2% [94].

### 4.3. Sensors of Small Molecules

**Figure 9:**
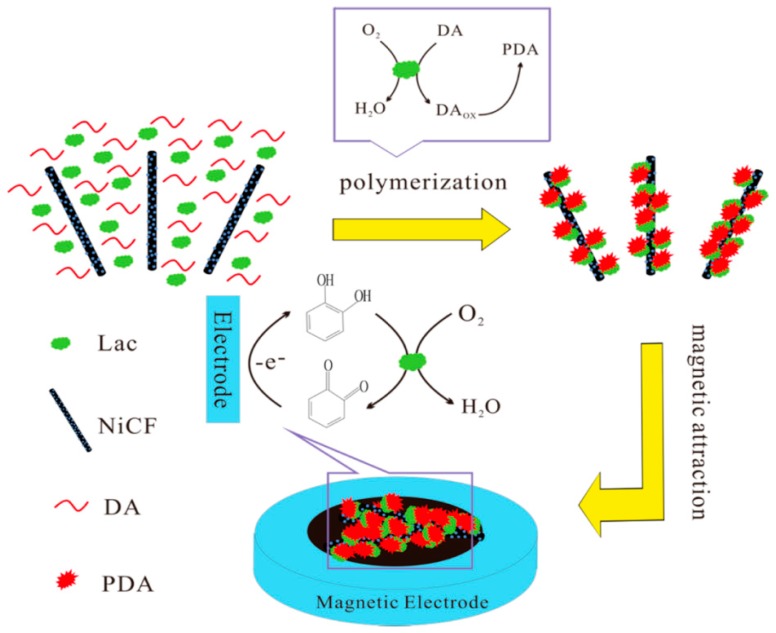
Synthetic route of magnetic Polydopamine-Laccase-Ni NP loaded CNFs composite and its catalytic oxidation of catechol on the electrode. Reprinted with permission from Reference [56]. Copyright American Chemical Society, 2014.

CNFs-based nanomaterials can not only be used to detect gas molecules and strain sensing, but can also to detect small molecules [96]. Table 1 lists the CNF-based nanomaterials for detecting different small molecules and their properties. Huang et al. loaded palladium NPs on CNFs to prepare a Pd/CNFs modified carbon paste electrode for the detection of dopamine (DA), uric acid (UA), and ascorbic acid (AA) [57]. After being modified with Pd NPs-loaded CNFs (Pd/CNFs), the oxidation overpotentials of DA, UA, and AA were significantly reduced when compared to the bare carbon paste electrode. The detection limits of Pd/CNFs modified carbon paste electrodes for DA, UA and AA were 0.2 μM, 0.7 μM, and 15 μM, respectively, and the linear range was 0.5–160 μM (DA), 2–200 mΜ (UA), and 0.05–4 mM (AA). Liu et al. reported another Pd NPs-loaded CNFs modified carbon paste electrode for oxalic acid detection, the detection limit of the as-prepared sensor for oxalic acid as low as 0.2 mM, and shows a linear range from 0.2 to 45 nM [59]. Liu et al. also prepared a Ni/CNFs composite electrode by electrospinning for glucose detection [63]. The as-prepared Ni/CNFs hybrid shows higher sensitivity towards glucose due to the electrocatalytic activity of the Ni NPs and the stability of the carbon electrode. In the absence of chloride poisoning, the detection limit of the Ni/CNFs composite electrode for glucose is 1 μM, with a linear range of 2 μM–2.5 mM (R = 0.9997). Li and coworkers synthesized a magnetic composite through one-pot polymerization of dopamine, laccase, and Ni NPs loaded CNFs (Figure 9). The as-prepared magnetic composite exhibited high selectivity towards catechol, and showed a linear range from 1 to 9100 μM, with a detection limit of 0.69 μM for catechol in water samples [56]. 

Lee et al. fabricated a ZnO/CNFs composite for the detection of DMMP, and ZnO NPs decorated on CNFs increased the specific surface area of the sensor and its affinity for DMMP [66]. The detection limit of ZnO/CNFs composite for DMMP is 0.1 ppb, with a linear range of 0.1–1000 ppb. Huang et al. modified glass carbon electrode using electrospun CNFs loaded with Ag-Pt alloy NPs [70]. The as-prepared composite electrode can detect DA in the presence of UA and AA, and the detection limit for DA is 0.11 μm, and the linear range is 10–500 μm. Tang et al. directly modified CNFs onto carbon paste electrode for determining amino acids [51]. The detection limit for the L-tryptophan (Trp), L-tyrosine (Tyr), and L-cysteine (Cys) was 0.1 μm, with linear ranges of 0.1–119 μM for Trp, 0.2–107 μM for Tyr, and 0.15–64 μM for Cys. Li et al. prepared CuCo alloy NPs-decorated CNFs by electrospinning [72]. The as-prepared CuCo/CNFs composite exhibits high sensitivity to glucose in human urine. The response time for glucose is 2 s and the linear range is 0.02–11 mM.

### 4.4. Sensors of Biomacromolecules

The high surface area and large number of active sites of CNFs can not only provide the grounds for the adsorption of proteins and enzymes, but CNFs can also provide the direct electron transfer and stabilize enzyme activity [103]. Therefore, CNFs are the most promising substrates for the development of biosensors [104,105,106]. Periyaruppan and coworkers developed a CNF-based nanoelectrode array for cardiac troponin-I (cTnI) detection in the early diagnosis of myocardial infraction [49]. After being modified with the anti-cTnI, the as-prepared biosensor showed high selectivity and sensitivity to cTnI, it could detect as low as 0.2 ng/mL of cTnI, and showed linear concentration relationships in the ranges of 0.25–1.0 and 5.0–100 mg/mL.

**Figure 10:**
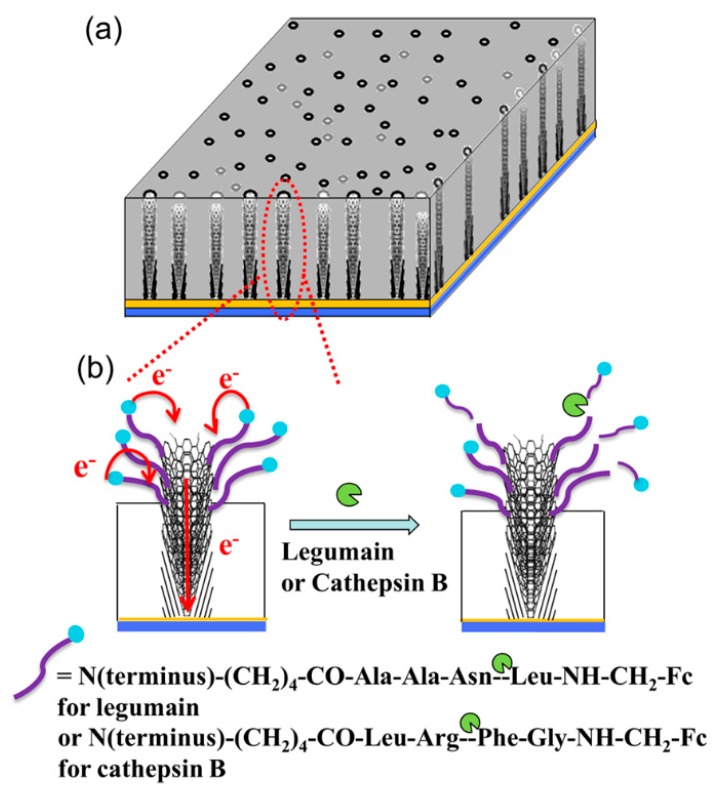
(**a**) Vertically aligned carbon nanofiber (VACNF) array embedded in the SiO_2_ matrix and (**b**) electron transfer from appended ferrocene at the distal end of the peptide to the underlying metal film electrode through the VACNFs and the loss of the electrochemical signal from ferrocene due to the cleavage of the peptide at specific sites. Reprinted with permission from Reference [109]. Copyright American Chemical Society, 2013.

In order to protect the protein from protease attack, Vamvakaki et al. used biomimetically synthesized silica to encapsulate the CNFs-immobilized enzyme acetylcholine esterase [74]. The obtained silica/CNF architecture improves enzyme stabilization against thermal denaturation and avoids protease attack, and exhibits an operational lifetime of more than 3.5 months under continuous polarization. Arumugam and coworkers fabricated a 3 × 3-array biosensor using nanopatterned vertically aligned CNF arrays (VACNFs) for E. Coli O157:H7 detection, the as-prepared patterned array showed nanoelectrode behavior and produced reliable electrochemical responses with high signal-to-noise (˃3) [107]. Gupta et al. also reported a nanoelectrode array based on vertically aligned CNFs, and found that the decrease in redox current and the increase in charge transfer resistance are proportional to the concentration of the C-reactive protein [108]. The detection limit of this biosensor for C-reactive protein reaches 90 pM, which is in the clinically relevant range. Later, Swisher and coworkers fabricated another nanoelectrode arrays using VACNFs for measuring the activity of proteases [109]. As shown in Figure 10, legumain and cathepsin B are covalently attached to the exposed VACNFs tip, with a ferrocene moiety linked at the distal end. The enhanced AC voltammetry properties enable the kinetic measurements of proteolytic cleavage of the surface-attached tetrapeptides by proteases, and the “specificity constant” k_cat_/K_m_ of the VACNF nanoelectrode arrays for cathepsin B and legumain is (4.3 ± 0.8) × 10^4^ and (1.13 ± 0.38) × 10^4^ M^−1^ s^−1^, respectively. These values are about two times that measured by a fluorescence assay.

## 5. Conclusions and Outlooks

Based on the above introduction and discussion on the synthesis and sensor applications of CNF-based functional nanomaterials, it can be concluded that CNFs play important roles for the fabrication of various sensors for gas, pressure, strain, small molecules, macromolecules, and other analytes. The using of CNF-based materials for sensor applications has a few advantages, for instance, the mesoporous of CNFs and nano/micro porous structures of CNF-based materials improved the surface area of electrode materials, the modification of CNFs with various NPs, polymers, and biomolecules enhanced the sensing performance, and the physical and chemical interactions between analytes and CNFs increased the sensing sensitivity of the fabricated sensors. Moreover, CNFs can be continuously prepared by electrospinning and raw materials polyvinylpyrrolidone (PVP) and polyacrylonitrile (PAN) are inexpensive. CNFs-based sensors generally exhibit high stability and selectivity to target molecules due to the high mechanical strength and chemical inertness of CNFs, and its ability to significantly reduce the oxidation overpotential. It is believed that this work will be valuable for readers to develop novel CNF-based functional materials through various fabrication techniques, and explore other potential applications in energy, catalysis, and environmental science.

While the synthesis and applications of CNFs and CNF-based materials have been widely studied in the last years, in our opinion, more efforts could be done in the following aspects. First, new synthesis methods for CNFs could be developed. Currently, chemical vapor deposition and electrospinning are the main strategies for creating CNFs. Other methods like template-based synthesis, self-assembly, and chemical hydrothermal methods could also be considered to achieve in the synthesis of CNFs with high efficiency. Second, the biological modification of CNFs for subsequent biomedical applications including biosensors, anti-bacterial materials, bone tissue engineering, and others could be further explored. Third, it is possible to fabricate CNF-based of 2D membranes and 3D aerogels/scaffolds for water purification applications. In addition, more attentions could be paid to the design and fabrication of high-performance energy storage materials/devices such as batteries, supercapacitors, solar cells, and fuel cells by introducing suitable functional nanoscale building blocks into the CNF systems.

## Figures and Tables

**Table 1:** Different CNF-based nanomaterials for small molecules detection ^1^.

Sensor	Target Molecule	Linear Range	Detection Limit	Reference
CNFs	DA	0.2–700,000 μM	0.08 μM	[97]
Pd/CNFs	H_2_O_2_ and NADH	0.2–20,000 μM (H_2_O_2_), 0.2–716.6 μM (NADH)	0.2 μM(H_2_O_2_)	[57]
Pd/CNFs	DA, UA, AA	0.5–160 μM (DA), 2–200 mΜ (UA), 0.05–4 mM (AA)	0.2 μM (DA), 0.7 μM(UA), 15 μM(AA)	[57]
Ni/CNFs	Glucose	2–2500 μM	1 μM	[63]
PANI-IL-CNF	Phenol	40–2100 nM	0.1 nM	[98]
Pd/CNFs	OA	0.2–45 mM	0.2 mM	[59]
Pt/CNFs	Glucose	2–20 mM		[64]
Rh/CNFs	Hydrazine	0.5–175 μM	0.3 μM	[61]
CNFs	Xanthine	0.03–21.19 μM	20 nM	[50]
ZnO/CNFs	DMMP	0.1–1000 ppb	0.1 ppb	[66]
Pt/CNFs	H_2_O_2_	1–800 μM	0.6 μM	[53]
GNPs/CNF/Au	CC and HQ	5–350 μM (CC), 9–500 μM (HQ)	0.36 μM (CC), 0.86 μM (HQ)	[99]
MCNF/PGE	DA, UA, AA	0.05–30 μM (DA), 0.5–120 μΜ (UA), 0.1–10 mM (AA)	0.02 μM (DA), 0.2 μM(UA), 50 μM(AA)	[46]
CNFs	Trp, Tyr, Cys	0.1–119 μM (Trp), 0.2–107 μM (Tyr), 0.15–64 μM (Cys)	0.1 μM	[51]
CNFs	CC, HQ	1–200 μM	0.2 μM (CC), 0.4 μM (HQ)	[52]
VACNFs	DA, 5-HT	1–10 μM	50 nM (DA), 250 nM (5-HT)	[48]
CuO/rGO/CNFs	Glucose	1–5.3 mM	0.1 μM	[100]
Pd-HCNF	H_2_O_2_, Glucose	5–2100 μM (H_2_O_2_), 0.06–6 mM (glucose)	3 μM(H_2_O_2_), 0.03 mM (glucose)	[54]
HRP-CNFs	H_2_O_2_	1–10 μM	1.3 μM	[101]
PtNP-CNF	H_2_O_2_	25–1500 μM	11 μM	[55]
Co_3_O_4_/CNFs	H_2_O_2_	1–2580 μM	0.5 μM	[102]
Ag-Pt/pCNFs	DA	10–500 μM	0.11 μM	[70]
Cu/CNFs	Catechol	9.95–9760 μM	1.18 μM	[62]
PDA-Lac-NiCNFs	Catechol	1–9100 μM	0.69 μM	[56]
Pd-Ni/CNFs	Sugar	0.03–800 μM	7-20 nm	[71]
CuCo-CNFs	Glucose	0.02–11 mM	1 μM	[72]

^1^ CNFs: carbon nanofibers; DA: dopamine; Pd/CNFs: palladium nanoparticle-loaded CNFs; NADH: nicotinamide adenine dinucleotide; UA: uric acid; AA: ascorbic acid; Ni/CNFs: Ni NP-loaded CNFs; PANI-IL-CNF: polyaniline-ionic liquid-CNF; OA: oxalic acid; Pt/CNFs: platinum NP-loaded CNFs; Rh/CNFs: rhodium NP-loaded CNFs; DMMP: dimethyl methylphosphonate; ZnO/CNFs: ZnO decorated CNFs; GNPs/CNF/Au: gold electrode modified with CNFs and gold NPs; CC: catechol; HQ: quinone; MCNF/PGE: mesoporous CNF-modified pyrolytic graphite electrode; Trp: L-tryptophan; Tyr: L-tyrosine; Cys: L-cysteine; VACNFs: vertically aligned CNFs; 5-HT: serotonin; CuO/rGO/CNFs: CuO nanoneedle/reduced graphene oxide/CNFs; Pd-HCNF: palladium-helical CNF hybrid; HRP-CNFs: CNFs modified with horseradish peroxidase; PtNP-CNF: platinum NP-decorated CNF; Ag-Pt/pCNFs: nanoporous CNFs decorated with Ag-Pt bimetallic NPs; Cu/CNFs: copper/carbon composite nanofibers; PDA-Lac-NiCNFs: polydopamine-laccase-nickel NP loaded CNFs; Pd-Ni/CNFs: Pd-Ni alloy NP/CNF composites; CuCo-CNFs: bimetallic CuCo NPs anchored and embedded in CNFs.
